# Associations of Growth Impairment and Body Composition among South African School-Aged Children Enrolled in the *KaziAfya* Project

**DOI:** 10.3390/nu13082735

**Published:** 2021-08-09

**Authors:** Kurt Z. Long, Johanna Beckmann, Christin Lang, Harald Seelig, Siphesihle Nqweniso, Nicole Probst-Hensch, Ivan Müller, Uwe Pühse, Peter Steinmann, Rosa du Randt, Cheryl Walter, Jürg Utzinger, Markus Gerber

**Affiliations:** 1 Swiss Tropical and Public Health Institute, CH-4002 Basel, Switzerland; nicole.probst@swisstph.ch (N.P.-H.); peter.steinmann@swisstph.ch (P.S.); juerg.utzinger@swisstph.ch (J.U.); 2 University of Basel, CH-4002 Basel, Switzerland; 3 Department of Sport, Exercise and Health, University of Basel, CH-4002 Basel, Switzerland; johanna.beckmann@unibas.ch (J.B.); christin.lang@unibas.ch (C.L.); harald.seelig@unibas.ch (H.S.); ivan.mueller@unibas.ch (I.M.); uwe.puehse@unibas.ch (U.P.); markus.gerber@unibas.ch (M.G.); 4 Department of Human Movement Science, Nelson Mandela University, Port Elizabeth 6001, South Africa; Felicitas.Nqweniso@mandela.ac.zamailto (S.N.); Rosa.durandt@mandela.ac.za (R.d.R.); cheryl.walter@nmmu.ac.za (C.W.)

**Keywords:** height, stunting, body composition, fat mass, school-aged children, South Africa

## Abstract

(1) Background: Early childhood malnutrition may result in increased fat mass (FM) among school-aged children in low- and middle-income countries (LMICs). We explored whether South African children with shorter stature have greater overall and abdominal FM compared to normal stature children. (2) Methods: Baseline assessments of body composition and weight were determined among school-aged children enrolled in a randomized controlled trial in Port Elizabeth, South Africa, using bioelectrical impedance analysis. Multiple linear regression models tested associations of children’s height and degree of stunting with FM, fat free mass (FFM), truncal fat mass (TrFM), and truncal fat free mass (TrFFM) overall and by sex. (3) Results: A total of 1287 children (619 girls, 668 boys) were assessed at baseline. Reduced child height was associated with higher FM and lower FFM and TrFFM, but these associations were reversed with increases in height. Girls classified as mildly or moderately/severely stunted had higher FM and TrFM but lower FFM and TrFFM, while no association was found for boys. (4) Conclusions: Our study suggests that efforts to reduce the non-communicable disease burden in LMICs should target growth-impaired children who may have greater overall FM and greater abdominal FM.

## 1. Introduction

The rapid increase in the global prevalence of overweight and obesity has become one of the most important contemporary health issues since it has led to a simultaneous worldwide increase in non-communicable diseases (NCDs) [1]. These increases are occurring as well in African populations with overweight/obesity and chronic, lifestyle-related diseases, contributing proportionately more to the burden of disease in these regions [2,3]. Similarly, African children are becoming overweight or obese, potentially predisposing them to NCDs in early adulthood even while they continue to be affected by infectious diseases [4].

Studies in several low- and middle-income countries (LMICs) have reported associations between impaired growth, obesity, and increased abdominal fat among adolescents and adults [5,6]. Stunted children may have excess body fat gain [7], increased abdominal fat [8,9], and higher truncal fat mass (TrFM) [10]. Children recovering from malnutrition have been found to have a disproportionately greater replenishment of body fat stores than lean body mass under conditions of adequate or excess energy intake [7,11,12]. Stunted children have also been found to have higher resting respiratory quotient (RQ) and impaired fat oxidation compared to their healthy, non-stunted peers [13]. These underlying metabolic changes and resulting accumulated fat mass (FM) associated with malnutrition in early childhood may increase the long-term risk of obesity and chronic metabolic disease in adulthood [14,15]. These associations are one form of the double burden of malnutrition (DBM) defined as the simultaneous co-occurrence of both undernutrition and overweight or obesity, and are now prevalent in many LMICs [16,17].

Studies have found inconsistent associations between measures of growth impairment and body composition, which may result from the different techniques used to assay body composition that are not comparable. However, studies using skinfold measures and/or waist girth in India and Senegal have found associations of stunting with increased FM or distribution of FM [8,18], while no associations were found among children in Jamaica or the lowlands of Peru [19,20]. Similarly, comparable studies using bioelectrical impedance analysis (BIA) or dual-energy X-ray absorptiometry (DXA) have found no association among South African children [21,22], while studies in Brazil have reported that boys, but not girls, gained more FM than controls [23]. These disparate findings may also be due to differences in the ages of enrolled children and their growth patterns [24], as well as to differences in the design and sample size of the study. Additionally, changing dietary and physical activity patterns in LMICs that are occurring with urbanization may be contributing to this heterogeneity [25,26].

The current study is a baseline analysis of an ongoing longitudinal randomized, controlled trial, assessing the effect of physical activity and multi-micronutrient supplementation on South African school-aged children’s growth, health, and wellbeing in a peri-urban setting. This baseline assessment in conjunction with the results of the longitudinal trial will address the lack of consensus regarding the associations of child growth impairment and body composition. Focusing on this critical period will provide an understanding of how body composition patterns are affected by pre-adolescent growth and development. The specific objective of the study is to determine associations of growth impairment measures among studied children at baseline with their body composition estimates. It tests whether growth impaired children have greater FM and reduced fat free mass (FFM) compared to non-growth-impaired children. Understanding this relationship can aid in the development of more effective community-specific interventions that reduce the burden of childhood obesity and long-term metabolic disease in sub-Saharan countries that are rapidly urbanizing.

## 2. Materials and Methods

### 2.1. Participating Schools and Subjects

Children included in this analysis were part of a larger randomized, controlled trial assessing the effect of physical activity and multi-micronutrient supplementation on children’s growth, health, and wellbeing (the *KaziAfya* project) in three African countries [27]. Children in the South African arm of the study reported here were recruited from public schools in marginalized peri-urban neighborhoods of Port Elizabeth in the Eastern Cape Province in March 2019. Contact with schools was sought via the school principals who were informed about the objectives, procedures, and potential risks and benefits of the study. Schools were eligible for the study if they had facilities to implement physical education lessons, if they were not involved in any other research projects, and were not in an area where government nutrition interventions or helminthiasis control interventions were taking place during the study period.

Children aged between 6 and 12 years at baseline were eligible for inclusion in the project if they were attending grades 1 to 4; were not participating in any food/nutritional programmes; were not suffering from clinical conditions other than growth impairment, which prevent participation in physical activity; and not participating in other clinical trials. Children were excluded from data analyses (but not from the intervention) if they had congenital or acquired alteration of the gastro-intestinal tract, impairing absorption of the multi-micronutrient supplements, or had received regular vitamin and mineral supplements through food/nutritional programmes in the past 6 months.

Oral assent was sought for each child’s participation in addition to written informed consent from parents/guardians. Participation of children was voluntary and children could withdraw at any time without further obligations. Each child was assigned a unique identification number to ensure confidentiality. Children were then enrolled after a parent/guardian granted written informed consent.

Power calculations for the overall study indicated that a total sample of 1096 children was needed per study site (calculations based on G*power 3.1: f = 0.10, alpha error probability = 0.05, power = 0.80, number of groups = 12, number of measurements = 3). Assuming an overall dropout-rate of 20%, the targeted sample size was determined to be 1,320 children per country.

### 2.2. Body Composition Estimates and Anthropometric Measures

Data assessments took place at children’s schools before children in the different intervention arms began receiving the treatment interventions. The main assessments were performed with three classes (~120 learners) across 2 school days with assessment procedures the same across all schools.

Body composition estimates for FM, FFM, TrFM and TrFFM in kg were assessed via dual-frequency BIA using the MC-580 wireless body composition monitor (Tanita Corp., Tokyo, Japan), a lightweight portable scale that uses tetra-polar BIA with four electrodes at the feet. MC-580 uses equations validated for children by comparison with a four compartmental model. Similar monitors have demonstrated acceptable accuracy for estimating total body fat in children when compared with DXA [28]. The study did not directly validate the BIA truncal fat values with a more valid technique, such as DXA, and there are few published studies reporting such validation. However, preliminary results reported by Heymsfield (personal communication) suggest that BIA-generated truncal fat values are comparable to values generated by DXA.

Children wearing only light sport clothing were asked to stand barefoot on the metal plates of the machine, being guided by the research assistant to ensure optimal contact according to the device manufacturer’s instructions. The MC-580 was also used to assess body weight, to the nearest 0.1 kg. Body height was taken to the nearest 0.1 cm with each child standing with back erect and shoulders against a stadiometer. Sex-specific height, and weight-for-age and body mass index (BMI) z-scores were then computed from the Centers for Disease Control and Prevention (CDC)/World Health Organization (WHO) growth reference data [29]. Children with z-scores between −1 standard deviation (SD) > z > −2 SD for height-for-age z-score (HAZ) were classified as mildly stunted, children with z-scores between −2 SD > z > −3 SD were classified as moderately stunted, while children with z –scores < −3 SD were classified as severely stunted; these thresholds are commonly used to define mild, moderate, and severe stunting or underweight [30]. Children were also classified using the CDC standards as underweight (below the 5th percentile), normal weight (5th percentile up to the 84th percentile), overweight (85th to the 94th percentile), and obese (equal to or greater than the 95th percentile).

Information was collected from parents regarding infrastructure and housing characteristics (house type, number of bed rooms, type of toilet, access to indoor water, indoor toilet/bathroom, and electricity) and related to ownership of three durable assets (presence of a working refrigerator, washing machine, and car). All collected data were double-entered, validated, and merged into a single SPSS data file. Survey data assessed via paper and pencil questionnaire were scanned and entered automatically (using EvaSys software version 8.0 from Electric Paper Evaluationssysteme GmbH, Lüneburg, Germany).

### 2.3. Statistics

The study outcomes were baseline estimates of overall FM, lean body mass, TrFM, and truncal lean body mass. Descriptive statistics (mean and SD) were calculated to describe characteristics of the sample. Mixed linear regression models with random intercepts for school classes were then used to test associations of children’s height as a continuous variable with each of these separate overall and truncal body composition estimates. A quadratic term for height was then included in each model to test for nonlinear relationship between height and the different body composition estimates. Associations of mild or moderate/severe stunting with the separate overall and truncal body composition outcomes were then tested in separate regression analyses. Additional analyses were carried out separately for boys and girls for each of the outcomes in the models, including height and stunting after considerable sex differences were found for a number of the outcomes (see results section and Table 1).

The regression model with height as the independent variable was adjusted for children’s sex, age, and weight at baseline, and a composite household socioeconomic status (SES) measure, which was a sum of household possessions. Models of associations between mild and moderate/severe stunting with body composition were only adjusted for weight and SES since the z-scores used in classifying children were already sex and age adjusted. Information on household SES was missing for approximately 30% of children. Accordingly, a categorical variable for the SES measure was created with missing values categorized as zero, values below the median of summed processions classified as one, and values greater than the median classified as two. Models were adjusted for weight to distinguish body composition compartments as children with greater height had a higher body weight compared with children with the low-growth classes [24]. All statistical tests were performed using SPSS^®^ 26 (IBM Corporation; Armonk, NY, USA) with statistical significance defined as *p* < 0.05.

## 3. Results

### 3.1. Child Characteristics

**Figure 1:**
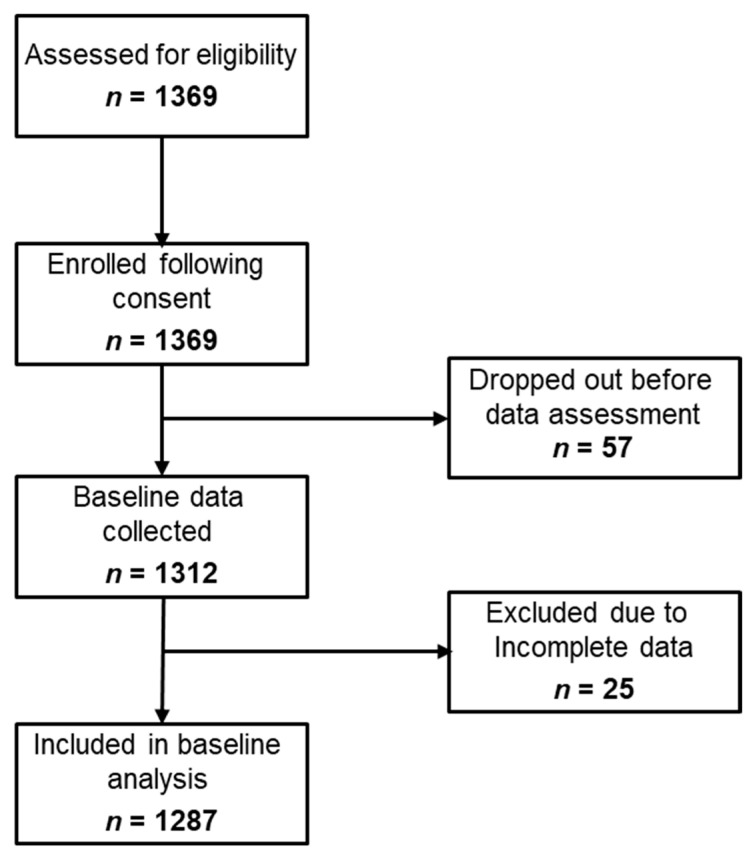
STROBE-nut flow diagram of children in the South African arm of the *KaziAfya* project included in the analysis.

A total of 1,369 children aged 6-12 years were enrolled (following written parental informed consent) from quintile three public schools in Port Elizabeth in the South African arm of the *KaziAfya* project. Fifty-seven children dropped out before the baseline assessment, while 25 children were excluded due to missing values for outcome variables or independent/confounding variables, leaving 1,287 that were included in this analysis (Figure 1). Approximately 52% of children were boys. Using the CDC standards, 4.7% of children were classified as underweight, 71% were normal weight, 10% were overweight, and 4.5% were obese.

A significantly greater percent of girls were overweight or obese compared to boys (Table 1). Approximately 30% and 8% of children were classified as mildly stunted or moderately/severely stunted, respectively. A greater proportion of boys were mildly stunted compared to girls, whereas no difference was found for moderate/severe stunting. Girls had greater FM and TrFM than boys (Table 1).

### 3.2. Associations of Growth Impairment with Overall FM and FFM

**Figure 2:**
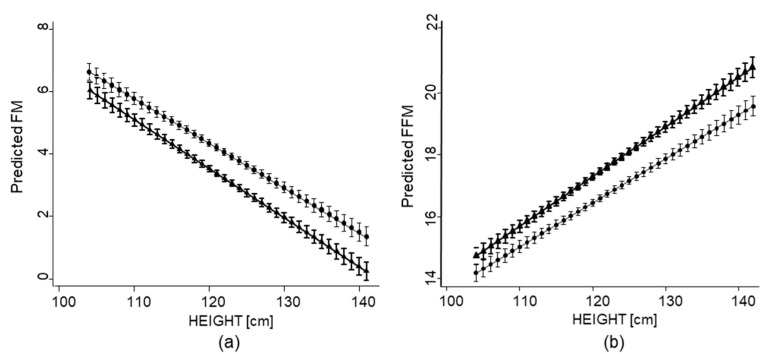
Association of height with body composition estimates by sex of school-aged children in the South African arm of the *KaziAfya* project: (**a**) FM; (**b**) FFM. Boy 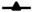; girl 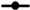.

The mixed linear regression model without a quadratic term provided the best fitting model. Height in this model was inversely associated with FM among all children combined in the multiple regression analyses after adjusting for child’s sex, age, and weight at baseline and SES (Table 2). These findings suggest that higher FM is associated with shorter stature among children but becomes incrementally lower with increases in children’s height. Similar associations were found separately among boys and girls as well. Girls, however, had significantly higher FM than boys for all measures of height (Table 2 and Figure 2).

In contrast, FFM was positively associated with height, suggesting that lower lean body mass is associated with shorter stature among children but becomes higher with increased height. Higher FFM among both boys and girls was also found to be positively associated with height. Girls had significantly lower FFM compared to boys across all measures of height (Table 2 and Figure 2).

**Figure 3:**
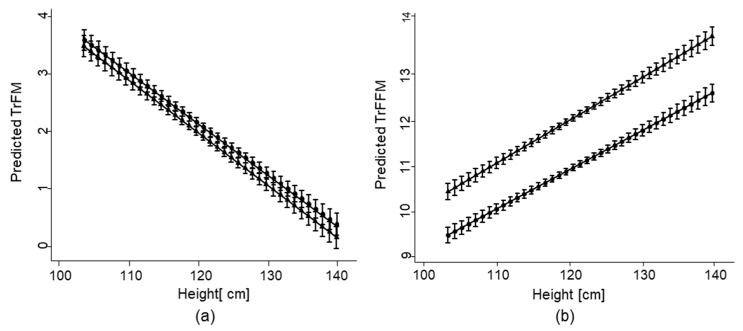
Association of height with body composition estimates by sex of school-aged children in the South African arm of the KaziAfya project: (**a**) TrFM; (**b**) TrFFM. Boy 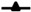; girl 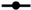.

Similar associations were found for TrFM and TrFFM. Height was again inversely associated with TrFM in the overall model, suggesting that children with reduced stature had higher abdominal FM, while children with greater height had higher lean body mass (Table 2). These associations were similar but not significantly different between both boys and girls (Table 2, Figure 3). A positive association between TrFFM and greater height was found in the overall analysis and again among boys and girls (Table 2, Figure 3). Girls have significantly lower FFM of approximately 1 kg compared to boys across all measures of height.

Moderate/severe stunting was associated with higher FM among all children compared to non-stunted children (Table 3). Mild and moderate/severe stunting were both associated with higher FM among girls but not among boys with the highest FM found among the more stunted girls. In contrast, children with moderate/severe stunting had lower FFM compared to non-stunted children. Lower FFM was also found among girls for each stunting category but again not among boys.

Higher TrFM was found among children with moderate/severe stunting similar to the pattern found for FM. Again, girls with both mild and moderate/severe stunting had higher TrFM compared to non-stunted girls, while no differences were found for boys. Finally, TrFFM was not associated with either category of stunting among all children. However, both mild and moderate/severe stunting was associated with lower TrFFM among girls but again not among boys.

## 4. Discussion

Studies in LMICs have reported associations between impaired growth, obesity, and increased FM among children and adolescents. These associations were examined among SouthAfrican school-aged children enrolled in a longitudinal school-based intervention trial to determine whether children with impaired growth are at greater risk of higher FM. We have found that baseline estimates of growth impairment among these South African children were associated with altered patterns of body composition and fat distribution. Children with reduced stature and classified as malnourished were found to have higher overall FM. Lean body mass, in contrast, was reduced among these children. Interestingly, girls with reduced stature had higher abdominal FM and lower TrFFM, while no association was found for boys. It is not possible, at this point, to infer causality in these associations given the cross-sectional design of the study. However, the finding of higher FM among children with moderate/severe growth impairment can portend greater risk for diabetes, cardiovascular disease, and hypertension later in adulthood, especially in LMICs passing through the nutrition transition [31]. Public health efforts to reduce the burden of non-communicable diseases (NCDs) in LMICs passing through the nutrition transition should consider targeting children with reduced growth trajectories.

The associations of nutritional growth impairment with increased FM in children and reduced lean body mass have been described in previous studies carried out in different LMICs [6,7,32]. Young stunted children in India were found to have higher levels of total and central body fat than their non-stunted counterparts [18]. Stunted girls in Senegal had more subcutaneous fat on the upper part of the body (trunk or arms) than non-stunted girls [8]. Cross-sectional studies in Brazil found that stunted school-aged children were more obese and had greater abdominal circumferences than non-stunted children [7]. Stunted boys in Brazil and in Mexico with slower height gain had greater FM and lower FFM compared to non-stunted boys or boys with greater height gain [23,24]. No significant differences were found between stunted and non-stunted girls or among girls with different growth trajectories. These findings are in contrast to our results where differences were found among girls but not among boys although our study may not be comparable with these given differences in design.

A number of studies have found no associations between children’s growth impairment and increased FM. Among South African children living in urban communities, no association was found between stunting at the end of infancy and body composition in late childhood [22]. A cross-sectional study of two study populations of children in rural South Africa found inconsistent results when comparing body composition of stunted and non-stunted children as measured by BMI, triceps, and subscapular skinfolds, and waist circumference [33]. Children in Nepal who had been stunted at 2 years of age maintained a shorter height and had a lower BMI at 8 years of age compared to non-stunted children [34]. Stunting at 2 years of age was not found to be associated with greater FM among school-aged children in Jamaica, but was associated with greater centralization of fat [10]. A subsequent longitudinal cohort study following children in this same community found no association between growth impairment and body composition, but did find that stunted children who grew more rapidly during childhood had a higher BMI at age 17 years compared with those who grew less rapidly [19]. The lack of associations reported by studies using BMI as an indicator of FM must be interpreted with caution since BMI is associated with FM, but is not a measure of adiposity.

More generally, the heterogeneity in findings across the various studies may be due to differences in study design and techniques used to determine body composition. However, both the Brazilian study that reported an association between stunting and body composition [23] and the South African study that found no such association [22] followed children longitudinally and used BIA or DXA. Differences in ages of enrolled children between studies may also have contributed to such heterogeneity. Premenarchal and postmenarchal girls have important differences in body composition, which can confound associations between growth impairment and body composition within studies and introduce heterogeneity across studies [35]. Children enrolled in our study were between 6 and 12 years of age and so were a more homogeneous population in terms of growth and body composition. However, the sexual maturation and physical development of children was not assessed using the Tanner scale and so there may have been underlying differences in the onset of menarche and resulting changes in body composition among girls.

Little is understood about the underlying biological mechanisms and drivers linking growth patterns and later body composition. Catch-up growth following growth impairment among children in LMICs may lead to greater replenishment of body fat stores than lean body mass [11,12,36,37]. Fjeld et al. [12] reported a 42% increase in fat weight gain among malnourished children, double the expected mean body fat content in young children. Stunting is associated with impairment of fat oxidation, regulating energy balance, and fat oxidation [13,38], which is a risk factor for excess weight gain and greater storage of fat in adipose tissues [39,40]. Such effects of stunting on fat oxidation have been found to contribute to proportionately greater FM and reduced muscle tissue among growth-impaired North Korean refugee children [41]. Sawaya et al. [7] also reported that a high-fat diet resulted in greater body fat gains in stunted girls in Brazil than non-stunted girls.

The drivers of the rapid increase in the global prevalence of overweight and obesity involving changes in dietary patterns and reduced physical activity may also be contributing to DBM in children. The nutrition transition among many LMICs has led to shifts from traditional diets of whole foods, such as pulses and whole grains to an energy-dense and nutrient-poor diet composed of refined high fat intake, and processed foods [16]. This shift is due to the rapid growth of modern retailing of consumer packaged processed foods and beverages and the growing dominance of supermarkets and food manufacturers involved in food distribution [42]. Shifts have also occurred in children’s physical activity in LMICs, with children becoming more sedentary as countries become more urbanized [43]. Reduced physical activity of children has been shown to be associated with greater FM, especially among boys [44]. Increased availability and consumption of high energy and high fat foods when coupled with reduced physical activity may further contribute to proportionately greater FM and result in rapid weight gain among stunted children with impaired fat oxidation.

The associations between reduced stature and greater overall and abdominal FM, and reduced lean body mass in our study reflects the stage of the nutrition transition that the Eastern Cape region of South Africa is passing through. Childhood stunting still persists in the Eastern Cape Province and more generally in South Africa, despite the rapid nutrition and lifestyle transitions that have occurred in the last several decades [45,46]. Multi-panel surveys have found increasingly higher levels of child overweight/obesity in the Eastern Cape from 2008 to 2017, especially among lower SES households [47]. This trend may be partly attributable to greater access and consumption of cheap high-calorific processed foods and low intakes of vegetables in urban lower-income households [48]. Simultaneously, up to one third of children in South Africa are physically inactive some of the highest rates in the world [49,50]. These drivers, when coupled with the biological mechanisms leading to greater FM in stunted children, may have contributed to the DBM patterns found in our study. These drivers may be impacting girls more than boys since studies in other regions of South Africa have found that physical activity was higher in boys than girls [51]. No clear sex differences in dietary patterns have been reported to date. This difference may also be partly due to the more advanced sexual development of girls compared to boys among the age range of this sample, which can affect the fat stores.

A number of limitations of our study need to be considered. The results have limited generalizability since all children are from peri-urban settings and quintile three schools located in disadvantaged areas in the Port Elizabeth area. A different picture might emerge in rural settings or in wealthier student populations across South Africa. Another limitation as noted above may be the cross-sectional design of the study and so the associations reported here must be interpreted with caution. However, this study is meant as a baseline analysis of an on-going trial and not an endpoint in itself. The follow-up assessments at two additional time points will allow us to draw a more accurate picture of associations between growth and body composition through time and so test their causal relationship [27].

The validity of using the BIA estimates generated by the Tanita monitor to assess overall FM may be viewed as an additional limitation of the study [52]. However, a number of studies have established that these estimates are very accurate relative to methods such as DXA [28]. A recent study by van Zyl et al. [53] developed a new impedance-based equation for body composition among black preadolescent children in South Africa based on comparisons with DXA measures. Expressing results in body composition terms provided by these estimates has more biological relevance and context for readers. However, these equations need to be tested further before they can be used in a study such as ours. Truncal values generated by BIA for adults or children have not been directly compared with values generated by such technique, as DXA as has been done for total FM. BIA-generated truncal fat values for children have been shown to be equivalent to DXA-generated values in preliminary unpublished results (Heymsfield, personal communication). Measures of children’s waist circumference were not carried out in our study and so it was not possible to compare more traditional measures of central adiposity with our TrFM and TrFFM estimates. Efforts that are more systematic must be carried out in the future to validate these truncal BIA values in pediatric populations.

## 5. Conclusions

Our findings show that growth impairment is associated with greater overall FM, which has important implications for the long-term consequences of children’s health. Increased childhood adiposity, especially in the abdominal region, can lead to greater risk of obesity in adulthood, which is associated with an increased risk of chronic metabolic disease [14,15]. These co-occurring risks require intervention approaches that simultaneously prevent or reduce the risk of both nutritional deficiencies leading to underweight, wasting, and stunting, and problems of obesity and NCDs [54]. Double duty approaches involving interventions, programmes, and policies that address both nutrient deficiencies and overweight, obesity, and NCDs may offer such an approach for reducing DBM in South Africa, where it is prevalent [55]

## Figures and Tables

**Table 1:** Child and household characteristics in the Port Elizabeth, South African component of the *KaziAfya* project.

	Total (*n* = 1287)	Boys (*n* = 668)	Girls (*n* = 619)
**Anthropometric measures**	M (SD) ^a^	M (SD)	M (SD)
Age (years)	8.33 (1.5)	8.20 (1.42) ^f^	8.37 (1.55) ^f^
Height (cm)	124.69 (9.24)	125.17 (8.88) ^f^	124.02 (9.34) ^f^
Weight—(kg)	25.38 (6.87)	25.38 (6.38)	25.18 (7.05)
BMI (kg/m^2^)	16.07 (2.61)	16.01 (2.42)	16.13 (2.80)
**Categories of malnutrition**	*n* (%)	*n* (%)	*n* (%)
Overweight ^b^	123 (10%)	53 (8.5) ^f^	68 (11.6)
Obese ^c^	61 (4.5%)	17 (2.7) ^f^	43 (7.3) ^f^
Mild stunting ^d^	500 (36.7)	249 (37.3)	251 (40.7)
Moderate/severe stunting ^e^	112 (8.2)	48 (7.2) ^f^	64 (10.4) ^f^
**Body composition**	M (SD)	M (SD)	M (SD)
Overall FM (kg)	5.94 (3.20)	5.43 (2.88) ^f^	6.40 (3.13) ^f^
Overall FFM (kg)	19.44 (4.26)	19.95 (4.07) ^f^	18.78 (4.2) ^f^
Truncal FM (kg)	2.81 (1.48)	2.60 (1.28) ^f^	3.00 (1.60) ^f^
Truncal FFM (kg)	12.46 (2.05)	12.9 (1.78) ^f^	11.96 (2.15) ^f^
**SES characteristics**			
Overall SES index	3.01 (1.20)	2.00 (1.24)	3.04 (1.15)

^a^ Mean values with standard deviations. Mean values with equal letters are significantly different (*p* < 0.05). ^b^ Overweight: BMI z-score > +1 SD (85th to the 94th percentile); ^c^ Obese: > +2 SD (equal to or greater than 95th percentile); ^d^ Moderate stunting: HAZ score ≥ −2 SD & <−1 SD; ^e^ Stunting: HAZ < −2 SD.

**Table 2:** Associations of overall and truncal body composition estimates with height among South African school-age children enrolled in the *KaziAfya* project.

Outcome	Overall (*n* = 1287) ^a^		Girls (*n* = 619)		Boys (*n* = 668) ^b^	
	B-Coefficient (SE)	*p* Value	B-Coefficient (SE)	*p* Value	B-Coefficient (SE)	*p* Value
**FM (kg)**						
Height	−0.16 (0.01)	<0.01	−0.16 (0.01)	<0.01	−0.14 (0.01)	<0.01
**FFM (kg)**						
Height	0.15 (0.01)	<0.01	0.16 (0.06)	<0.01	0.15 (0.01)	<0.01
**TrFM (kg)**						
Height	−0.09 (0.05)	<0.01	−0.10 (0.01)	<0.01	−0.09 (0.05)	<0.01
**TrFFM (kg)**						
Height	0.08 (0.01)	<0.01	0.08 (0.01)	<0.01	0.08 (0.01)	<0.01

^a^ Random effect model adjusted for age, sex, child age, socio-economic status and weight. ^b^ Random effect model adjusted for age, child age, socio-economic status and weight.

**Table 3:** Associations of overall and truncal body composition measures with growth impairment among South African school-age children enrolled in the *KaziAfya* project.

	Overall (*n* = 1287) ^a^		Girls (*n* = 619) ^b^		Boys (*n* = 668) ^b^	
Outcome	B-Coefficient (SE)	*p* Value	B-Coefficient (SE)	*p* Value	B-Coefficient (SE)	*p* Value
**FM (kg)**						
Mild stunting ^c^	−0.09 (0.09)	0.38	0.27 (0.12)	0.02	0.08 (0.12)	0.94
Stunted ^d^	0.70 (0.15)	<0.01	0.88 (0.18)	<0.01	0.31 (0.22)	0.16
**FFM (kg)**						
Mild stunting	−0.08 (0.09)	0.36	−0.27 (0.12)	0.02	−0.10 (0.12)	0.94
Stunted	−0.70 (0.11)	<0.01	−0.90 (0.18)	<0.01	−0.31 (0.22)	0.10
**TrFM (kg)**						
Mild stunting	0.08 (0.05)	0.11	0.16 (0.08)	0.04	0.98 (0.07)	0.20
Stunted	0.42 (0.09)	<0.01	0.54 (0.11)	<0.01	0.25 (0.12)	0.04
**TrFFM (kg)**						
Mild stunting	0.48 (0.01)	0.35	−0.22 (0.07)	<0.01	−0.02 (0.07)	0.80
Stunted	0.07 (0.74)	0.93	−0.57 (0.11)	≤0.01	−0.22 (0.12)	0.08

^a^ Random effect model adjusted for age, sex, child age, socio-economic status and weight. ^b^ Random effect model adjusted for age, child age, socio-economic status and weight. ^c^ Mild stunting: HAZ > −2 SD ≤ −1 SD; ^d^ Stunted: HAZ < −2 SD. Reference group is non-stunted children.

## Data Availability

All data analyzed during the overall study will be included in the published articles and their supplementary information files.

## References

[1] Saklayen M.G. (2018). The global epidemic of the metabolic syndrome. Curr. Hypertens. Rep..

[2] Steyn K., Damasceno A., Jamison D.T., Feachem R.G., Makgoba M.W., Bos E.R., Baingana F.K., Hoffman K.J., Rogo K.O. (2006). Lifestyle and related risk factors for chroic diseases. Disease and Mortality in Sub-Saharan Africa.

[3] Murray C.J.L., Vos T., Lozano R., Naghavi M., Flaxman A.D., Michaud C., Ezzati M., Shibuya K., Salomon J.A., Abdalla S. (2012). Disability-adjusted life years (DALYs) for 291 diseases and injuries in 21 regions, 1990–2010: A systematic analysis for the Global Burden of Disease Study 2010. Lancet.

[4] Klingberg S., Draper C.E., Micklesfield L.K., Benjamin-Neelon S.E., van Sluijs E.M.F. (2019). Childhood obesity prevention in Africa: A systematic review of intervention effectiveness and implementation. Int. J. Environ. Res. Public Health.

[5] Doak C., Monteiro C., Popkin B. (1999). The coexistence of obesity and undernutrition in the same households is an emerging phenomena in lower income countries. FASEB J..

[6] Popkin B.M., Richards M.K., Montiero C.A. (1996). Stunting is associated with overweight in children of four nations that are undergoing the nutrition transition. J. Nutr..

[7] Sawaya Al Grillo L.P., Verreschi S.I., da Silva A.C., Roberts S.B. (1998). Mild stunting is associated with higher susceptibility to the effects of high fat diets: Studies in a shantytown population in São Paulo, Brazil. J. Nutr..

[8] Bénéfce E., Garnier D., Simondon K.B., Malina R.M. (2001). Relationship between stunting in infancy and growth and fat distribution during adolescence in Senegalese girls. Eur. J. Clin. Nutr..

[9] Schroeder D.G., Martorell R., Flores R. (1999). Infant and child growth and fatness and fat distribution in Guatemalan adults. Am. J. Epidemiol..

[10] Walker S.P., Gaskin P.S., Powell C.A., Bennett F.I. (2002). The effects of birth weight and postnatal linear growth retardation on body mass index, fatness and fat distribution in mid and late childhood. Public Health Nutr..

[11] Sawaya A.L., Roberts S. (2003). Stunting and future risk of obesity: Principal physiological mechanisms. Cad. Saúde Pública.

[12] Fjeld C.R., Schoeller D.A., Brown K.H. (1989). Body composition of children recovering from severe protein-energy malnutrition at two rates of catch-up growth. Am. J. Clin. Nutr..

[13] Hoffman D.J., Sawaya A.L., Verreschi I., Tucker K.L., Roberts S.B. (2000). Why are nutritionally stunted children at increased risk of obesity? Studies of metabolic rate and fat oxidation in shantytown children from São Paulo, Brazil. Am. J. Clin. Nutr..

[14] Smith S.A. (2003). Central role of the adipocyte in the insulin-sensitizing and cardiovascular risk modifying actions of the thiazolidinediones. Biochemie.

[15] Daniels S.R., Morrison J.A., Sprecher D.L., Khoury P., Kimball T.R. (1999). Association of body fat distribution and cardiovascular risk factors in children and adolescents. Circulation.

[16] Popkin B.M., Corvalan C., Grummer-Strawn L.M. (2020). Dynamics of the double burden of malnutrition and the changing nutrition reality. Lancet.

[17] Wells J.C., Sawaya A.L., Wibaek R., Mwangome M., Poullas M.S., Yajnik C.S., Demaio A. (2020). The double burden of malnutrition: Aetiological pathways and consequences for health. Lancet.

[18] Savanur M.S., Ghugre P.S. (2016). BMI, body fat and waist-to-height ratio of stunted v. non-stunted Indian children: A case-control study. Public Health Nutr..

[19] Walker S.P., Chang S.M., Powell C.A. (2007). The association between early childhood stunting and weight status in late adolescence. Int. J. Obes..

[20] Pomeroy E., Stock J.T., Stanojevic S., Miranda J.J., Cole T.J., Wells J.C. (2014). Stunting, adiposity, and the individual-level “dual burden” among urban lowland and rural highland Peruvian children. Am. J. Hum. Biol..

[21] Kagura J., Feeley A.B., Micklesfield L.K., Pettifor J.M., Norris S.A. (2012). Association between infant nutrition and anthropometry, and pre-pubertal body composition in urban South African children. J. Dev. Orig. Health Dis..

[22] Cameron N., Wright M.M., Griffiths P.L., Norris S.A., Pettifor J.M. (2005). Stunting at 2 years in relation to body composition a 9 years in African urban children. Obes. Res..

[23] Martins P.A., Hoffman D.J., Fernandes M.T., Nascimento C.R., Roberts S.B., Sesso R., Sawaya A.L. (2004). Stunted children gain less lean body mass and more fat mass than their non-stunted counterparts: A prospective study. Br. J. Nutr..

[24] Barrios P.L., Garcia-Feregrino R., Rivera J.A., Barraza-Villarreal A., Hernández-Cadena L., Romieu I., Gonzalez-Casanova I., Ramakrishnan U., Hoffman D.J. (2019). Height trajectory during early childhood is inversely associated with fat mass in later childhood in Mexican boys. J. Nutr..

[25] Swinburn B.A., Sacks G., Hall K.D., McPherson K., Finegood D.T., Moodle M.L., Gortmaker S.L. (2011). The global obesity pandemic: Shaped by global drivers and local environments. Lancet.

[26] Gerber M., Endes K., Herrmann C., Colledge F., Brand S., Donath L., Faude O., Pühse U., Hanssen H., Zahner L. (2017). Fitness, stress, and body composition in primary schoolchildren. Med. Sci. Sports Exerc..

[27] Gerber M., Ayekoé S.A., Beckmann J., Bonfoh B., Coulibaly J.T., Daouda D., du Randt R., Finda L., Gall S., Mollel G.J. (2020). Effects of school-based physical activity and multi-micronutrient supplementation intervention on growth, health and well-being of schoolchildren in three African countries: The KaziAfya cluster randomised controlled trial protocol with a 2 × 2 factorial design. Trials.

[28] Barreira T.V., Staiano A.E., Katzmarzyk P.T. (2013). Validity assessment of a portable bioimpedance scale to estimate body fat percentage in white and African-American children and adolescents. Pediatr. Obes..

[29] Ogden C.L., Kuczmarski R.J., Flegal K.M., Mei Z., Guo S., Wei R., Grummer-Strawn L.M., Curtin L.R., Roche A.F., Johnson C.L. (2002). Centers for Disease Control and Prevention 2000 growth charts for the United States: Improvements to the 1977 National Center for Health Statistics version. Pediatrics.

[30] Stevens G.A., Finucane M.M., Paciorek C.J., Flaxman S.R., White R.A., Donner A.J., Ezzati M. (2012). Trends in mild, moderate, and severe stunting and underweight, and progress towards MDG 1 in 141 developing countries: A systematic analysis of population representative data. Lancet.

[31] De Lucia Rolfe E., de França G.V.A., Avila Vianna C., Gigante D.P., Miranda J.J., Yudkin J.S., Lessa Horta B., Ong K.K. (2018). Associations of stunting in early childhood with cardiometabolic risk factors in adulthood. PLoS ONE.

[32] Sawaya A.L., Dallal G., Solymos G., de Sousa M.H., Ventura M.L., Roberts S.B., Sigulem D.M. (1995). Obesity and malnutrition in a Shantytown population in the city of São Paulo, Brazil. Obes. Res..

[33] Motswagole B.S., Kruger H.S., Faber M., Monyeki K.D. (2012). Body composition in stunted, compared to non-stunted, black South African children, from two rural communities. S. Afr. J. Clin. Nutr..

[34] Wells J.C.K., Devakumar D., Manandhar D.S., Saville N., Chaube S.S., Costello A., Osrin D. (2019). Associations of stunting at 2 years with body composition and blood pressure at 8 years of age: Longitudinal cohort analysis from lowland Nepal. Eur. J. Clin. Nutr..

[35] Vink E.E., van Coeverden S.C., van Mil E.G., Felius B.A., van Leerdam F.J., Delemarre-van de Waal H.A. (2010). Changes and tracking of fat mass in pubertal girls. Obesity.

[36] Martorell R., Khan L.K., Schroeder D.G. (1994). Reversibility of stunting: Epidemiological findings in children from developing countries. Eur. J. Clin. Nutr..

[37] Hills A., Byrne N.M. (2010). An overview of physical growth and maturation. Med. Sport Sci..

[38] Hoffman D.J., Roberts S.B., Verresch I., Martins P.A., de Nascimento C., Tucker K.L., Sawaya A.L. (2000). Regulation of energy intake may be impaired in nutritionally stunted children from the shantytowns of São Paulo, Brazil. J. Nutr..

[39] Astrup A., Buemann B., Christensen N.J., Toubro S. (1994). Failure to increase lipid oxidation in response to increasing dietary fat content in formerly obese women. Am. J. Physiol..

[40] Seidell J.C., Muller D.C., Sorkin J.D., Andres R. (1992). Fasting respiratory exchange ratio and resting metabolic rate as predictors of weight gain: The Baltimore Longitudinal Study on Aging. Int. J. Obes. Relat. Metab. Disord..

[41] Lee S.K. (2017). North Korean children: Nutrition and growth. Ann. Pediatr. Endocrinol. Metab..

[42] Popkin B.M. (2014). Nutrition, agriculture and the global food system in low and middle income countries. Food Policy.

[43] Ford N., Patel S.A., Narayan K.M.V. (2017). Obesity in low-and middle-income countries: Burden, drivers, and emerging challenges. Annu. Rev. Public Health.

[44] Ness A.R., Leary S.D., Mattocks C., Blair S.N., Reilly J.J., Wells J., Ingle S., Tilling K., Smith G.D., Riddoch C. (2007). Objectively measured physical activity and fat mass in a large cohort of children. PLoS Med..

[45] Said-Mohamed R., Micklesfield L.K., Pettifor J.M., Norris S.A. (2015). Has the prevalence of stunting in South African children changed in 40 years? A systematic review. BMC Public Health.

[46] Abrahams Z., McHiza Z., Steyn N.P. (2011). Diet and mortality rates in Sub-Saharan Africa: Stages in the nutrition transition. BMC Public Health.

[47] Sartorius B., Sartorius K., Green R., Lutge E., Scheelbeek P., Tanser F., Dangour A.D., Slotow R. (2020). Spatial-temporal trends and risk factors for undernutrition and obesity among children (<5 years) in South Africa, 2008–2017: Findings from a nationally representative longitudinal panel survey. BMJ Open.

[48] McLachlan M., Landman A.P. (2013). Nutrition-sensitive agriculture—A South African perspective. Food Secur..

[49] De Vos J.C.W., Du Toit D., Coetzee D. (2016). The types and levels of physical activity and sedentary behaviour of senior phase learners in Potchefstroom. Health S. Afr. Gesondheld.

[50] Gerber M., Müller I., Walter C., du Randt R., Adams L., Gall S., Joubert N., Nqweniso S., Smith D., Steinmann P. (2018). Physical activity and dual disease burden among South African primary schoolchildren from disadvantaged neighbourhoods. Prev. Med..

[51] Micklesfield L.K., Pedro T.M., Kahn K., Kinsman J., Pettifor J.M., Tollman S., Norris S.A. (2014). Physical activity and sedentary behavior among adolescents in rural South Africa: Levels, patterns and correlates. BMC Public Health.

[52] Jebb S.A., Cole T.J., Doman D., Murgatroyd P.R., Prentice A.M. (2000). Evaluation of the novel Tanita body-fat analyser to measure body composition by comparison with a four-compartment model. Br. J. Nutr..

[53] Van Zyl A., White Z., Ferreira J., Wenhold F.A.M. (2019). Developing an impedance based equation for fat-free mass of Black preadolescent South African children. Nutrients.

[54] Hawkes C., Ruel M.T., Salm L., Sinclair S., Branca F. (2020). Double-duty actions: Seizing programme and policy opportunities to address malnutrition in all its forms. Lancet.

[55] WHO (2017). Double-Duty Actions.

